# Supplementary material for: The interactome of intact mitochondria by cross-linking mass spectrometry provides evidence for coexisting respiratory supercomplexes
Source: Mol Cell Proteomics. 2017 Dec 8;17(2):216–32. doi: 10.1074/mcp.RA117.000470 (PMC5795388; doi:10.1074/mcp.RA117.000470)
Supplement: Supplemental Data [file supp_RA117.000470_133922_0_supp_23978_fzffwf.zip › spectra_annotation/mito_DR_spectra_annotation/221-1-1-1-5-1.pdf]

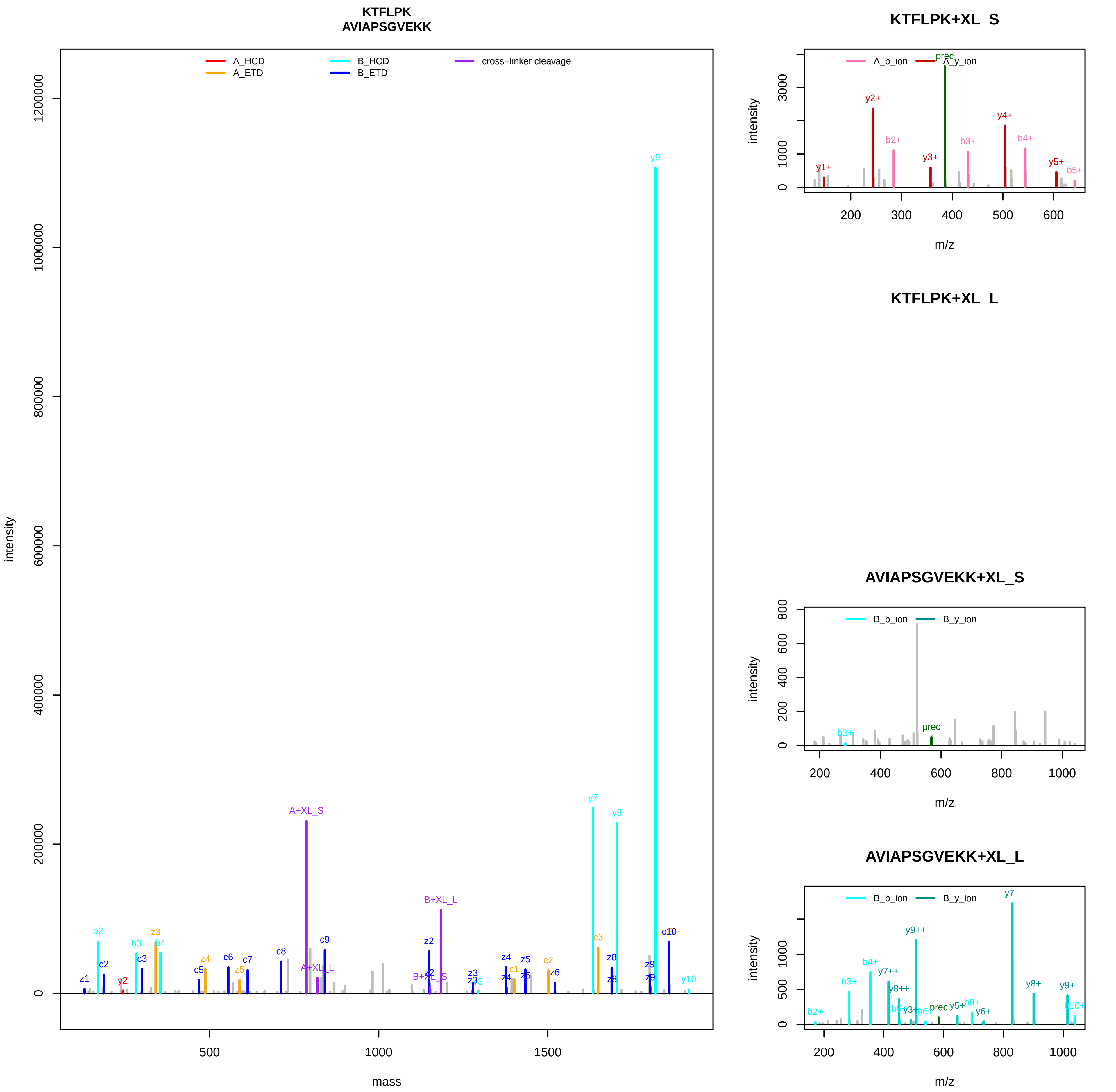
KTFLPK
AVIAPSGVEKK
A_HCD
A_ETD
B_HCD
B_ETD
cross−linker cleavage
intensity
mass
0
200000
400000
600000
800000
1000000
1200000
500
1000
1500
z1 b2 c2 y2 b3 c3 z3 b4 c5 z4 c6 z5 c7 c8 A+XL_S A+XL_L c9 B+XL_S z2 B+XL_L z3 z4 c1 z5 c2 z6 y7 c3 y8 z8 z9 y9 c10 y10
KTFLPK+XL_S
A_b_ion
A_y_ion
prec
y1+ y2+ b2+ y3+ b3+ y4+ b4+ y5+ b5+
intensity
m/z
0
1000
3000
200
300
400
500
600
KTFLPK+XL_L
AVIAPSGVEKK+XL_S
B_b_ion
B_y_ion
b3+
prec
intensity
m/z
0
200
400
600
800
1000
AVIAPSGVEKK+XL_L
B_b_ion
B_y_ion
b2+ b3+ b4+ y7++ y8++ y9++ y3+ b6+ prec y5+ b8+ y6+ y7+ y8+ y9+ b10+
intensity
m/z
0
500
1000
200
400
600
800
1000
